# Comparison of Mutation Profiles in the Duchenne Muscular Dystrophy Gene among Populations: Implications for Potential Molecular Therapies

**DOI:** 10.3390/ijms16035334

**Published:** 2015-03-09

**Authors:** Luz Berenice López-Hernández, Benjamín Gómez-Díaz, Alexandra Berenice Luna-Angulo, Mónica Anaya-Segura, David John Bunyan, Carolina Zúñiga-Guzman, Rosa Elena Escobar-Cedillo, Bladimir Roque-Ramírez, Luis Angel Ruano-Calderón, Héctor Rangel-Villalobos, Julia Angélica López-Hernández, Francisco Javier Estrada-Mena, Silvia García, Ramón Mauricio Coral-Vázquez

**Affiliations:** 1 National Medical Centre “20 de Noviembre”, Institute for Social Security of State Workers, Mexico City 03100, Mexico; E-Mails: bladimir_roque@hotmail.com (B.R.-R.); rolasil@yahoo.com.mx (S.G.); 2 Asociación de Distrofia Muscular de Occidente A.C., Guadalajara 44380, Mexico; E-Mails: monica207383614@gmail.com (M.A.-S.); anilorac_zg911@hotmail.com (C.Z.-G.); 3 National Institute of Rehabilitation, Mexico City 14389, Mexico; E-Mails: bgodiaz@gmail.com (B.G.-D.); rescobarmex@gmail.com (R.E.E.-C.); 4 Department of Molecular Biology, Panamerican University, Mexico City 03920, Mexico; E-Mails: lunangulo@gmail.com (A.B.L.-A.); festrada@up.edu.mx (F.J.E.-M.); 5 University Center of Exact Sciences and Engineering, University of Guadalajara, Guadalajara 44430, Mexico; 6 Wessex Regional Genetics Laboratory, Salisbury District Hospital, Salisbury SP2 8BJ, UK; E-Mail: Dave.Bunyan@salisbury.nhs.uk; 7 General Hospital of Durango, Durango, 34000, Mexico; E-Mail: laruanoc@hotmail.com; 8 Instituto de Investigación en Genética Molecular, Centro Universitario de la Ciénega, Universidad de Guadalajara, Ocotlán, 47810, México; E-Mail: hrangel13@hotmail.com; 9 Department of Human Genetics, Leiden University Medical Center, Leiden 2333 ZA, The Netherlands; E-Mail: julyberries@hotmail.com; 10 Studies Section of Postgraduate and Research, School of Medicine, National Polytechnic Institute, Mexico City 11340, Mexico; E-Mail: rmcoralv@gmail.com

**Keywords:** Ataluren, DMD gene, MLPA, exon skipping, Duchenne, therapies

## Abstract

Novel therapeutic approaches are emerging to restore dystrophin function in Duchenne Muscular Dystrophy (DMD), a severe neuromuscular disease characterized by progressive muscle wasting and weakness. Some of the molecular therapies, such as exon skipping, stop codon read-through and internal ribosome entry site-mediated translation rely on the type and location of mutations. Hence, their potential applicability worldwide depends on mutation frequencies within populations. In view of this, we compared the mutation profiles of the populations represented in the DMD Leiden Open-source Variation Database with original data from Mexican patients (*n* = 162) with clinical diagnosis of the disease. Our data confirm that applicability of exon 51 is high in most populations, but also show that differences in theoretical applicability of exon skipping may exist among populations; Mexico has the highest frequency of potential candidates for the skipping of exons 44 and 46, which is different from other populations (*p* < 0.001). To our knowledge, this is the first comprehensive comparison of theoretical applicability of exon skipping targets among specific populations.

## 1. Introduction

**Figure 1:**
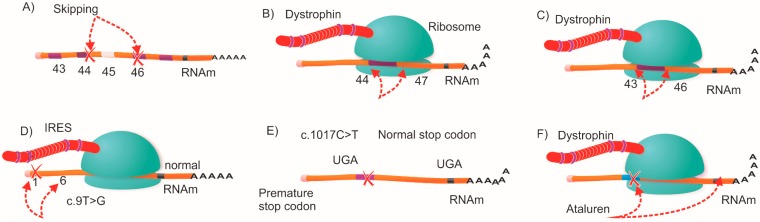
Molecular therapeutic strategies to restore dystrophin expression in Duchenne Muscular Dystrophy (DMD) patients. Reading frame restoration could be achieved in different combinations; (**A**) for an exon 45 deletion, the reading frame could be restored by targeted skipping of exons 44 or 46; while (**B**) and (**C**) show other in-frame combinations that allow the production of shorter dystrophins; (**D**) the IRES-induced translation mechanism; alternative initiation codon in exon 6 rescues dystrophin production (**E**) and (**F**) show the ribosomal read-through of a premature stop codon by the therapeutic agent Ataluren.

Duchenne Muscular Dystrophy (DMD; MIM# 310200) is a severe neuromuscular disease that causes disability and early death due to progressive muscle loss. Advances in molecular diagnosis [1,2] and patient management [3] have resulted in extended survival for patients [4]. DMD is caused by the lack of functional dystrophin molecules, either due to nonsense mutations in the *DMD* gene (premature stop codons) or by large rearrangements (deletions or duplications) that disturb the reading frame of the dystrophin gene and in consequence abolish the production of dystrophin in muscles [5]. Becker Muscular Dystrophy (BMD; MIM# 300376) is a mild form of the disease [6] in which internally-truncated dystrophin molecules are produced as result of in-frame deletions or duplications; these incomplete but functional proteins ameliorate the phenotype. Dystrophin is thought to serve as a shock absorber protein to protect muscle cells from movement-induced damage [7]. Novel therapeutic strategies are in development for DMD, either involving the replacement of the gene (cell-based therapies), exogenous delivery of functionally engineered dystrophin gene constructs (gene therapy) or by repairing the endogenous locus [8]—this involves strategies such as stop codon read-through [9] (using Translarna^®^/Ataluren, PTC Therapeutics, South Plainfield, NJ, USA), exon skipping (using Eteplirsen, Sarepta Therapeutics, Cambridge, MA, USA, or Drisapersen, Prosensa, Leiden, The Netherlands) (Figure 1) [10] or the recently described internal ribosome entry site (IRES)-induced translation [11].

Exon skipping (ES) and stop codon read-through recently gained interest because of the optimistic results in clinical trials [12,13,14]. ES aims to modify the splicing of pre-mRNA dystrophin transcripts converting the severe DMD phenotype into the milder BMD by inducing the production of incomplete but functional dystrophins [15]. Where DMD is due to a nonsense mutation, compounds such as Gentamicin and Ataluren may allow ribosomal read-through of the premature stop codons in dystrophin mRNA. Results of a phase II study of Ataluren showed a moderate clinical benefit in DMD patients [16,17]. These gene repair strategies are dependent on the type of mutation; therefore, a complete genetic screening that allows the exact characterization of the mutation is of utmost importance for future personalized medicine. The location and type of mutation will determine the best targets for exon skipping and may differ among populations. Herein we compare the frequencies of applicable exon skipping targets among different populations, including original data from Mexican-Mestizos, which comprise most of the present day Mexican population (~90%) [18].

## 2. Results and Discussion

### 2.1. Mutation Detection

**Figure 2:**
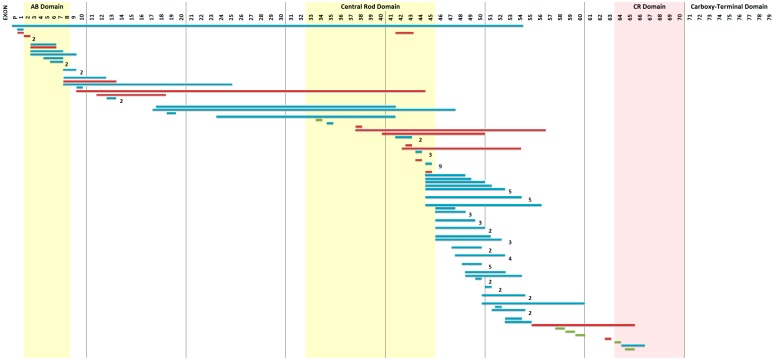
The mutation profile of Mexican-Mestizo patients. The horizontal bars represent the exons involved in mutations: deletions (**blue**), duplications (**red**) and point mutations (**green**).

Using the Point Mutation Multiplex Ligation-dependent Probe Amplification (PM-MLPA) [2] and High Resolution Melting (HRM) techniques, deletions or duplications were found in 105 of the 162 unrelated DMD patients (see Figure 2) and point mutations were present in another six cases (see Table 1) that represent 10.52% of all deletion/duplication negative cases. The overall mutation detection rate in DMD cases was 68.52%. Nine of the DMD cases had a novel mutation (see Table 2). From the BMD cohort (*n* = 10), deletions or duplications were found in five unrelated cases: three with a deletion of exons 45–47; (ex45ex47del→c.6439−?_6912+?del), one with a deletion of exons 45–49 (ex45ex49del→c.6439-?_7200+?del), and one with a duplication of exons 3–9 (ex03ex09dup→c.94-?_960+?dup). 

**Table 1:** Point mutations found in Mexican-Mestizo DMD patients.

Exon	Change	Stop Codon	Reported in LEIDEN Database
30	c.4120G > T p.(Glu1374Ter)	UAG	Novel
34	c.4693C > T p.(Gln1565Ter)	UAG	4 times
59	c.8713C > T p.(Arg2905Ter)	UGA	20 times
64	c.9337C > T p.(Arg3113Ter)	UGA	14 times
65	c.9380C > G p.(Ser3127Ter)	UGA	8 times
70	c.10171C > T p.(Arg3391Ter)	UGA	29 times

**Table 2:** Novel mutations found in Mexican-Mestizo DMD patients.

Involved Exon(s)/Change	Prediction
ex01ex54del→c.(?_-244)_8027+?del	No mRNA produced
ex65ex66del→c.9362-?_9649+?del	IN-FRAME duplication
ex10ex44dup→c.961-?_6438+?dup	IN-FRAME duplication
ex38ex56dup→c.5326-?_8390+?dup	OUT-OF-FRAME duplication
ex41ex50dup→c.5740-?_7309+?dup	OUT-OF-FRAME duplication
ex43ex54dup→c.6118-?_8027+?dup	OUT-OF-FRAME duplication
ex56ex65dup→c.8218-?_9563+?dup	OUT-OF-FRAME duplication
ex63dup→c.9225-?_9286+?dup	OUT-OF-FRAME duplication
ex 30 c.4120G > T p.(Glu1374Ter)	Stop codon

### 2.2. Genotype-Phenotype Correlation

DMD is usually caused by mutations that disrupt the reading frame; 89.5% (*n* = 94) of our patients followed this rule, but eleven out of 105 (10.5%) cases with clinical features of DMD had in-frame deletions or duplications that would be anticipated to result in BMD. For the BMD cohort, all five cases with a known mutation had in-frame deletions or duplications, so mutations in this group were consistent with the expected phenotype (BMD).

### 2.3. Theoretical Applicability of Exon Skipping and Stop Codon Read-Through among Populations 

Comparison of applicable exon skipping targets among populations was performed using data from the DMD Leiden Open-source Variation Database (LOVD). A inter and intra population comparison was performed. The search filters used were: country, phenotype = DMD and technique = MLPA, and the combinations of deletions/duplications (single and multiple exon mutations) in which the reading frame could be restored with the unique omission of exons 44, 45, 46, 51 and 53 (see Figure 1A–C) [19]. Our inter-population analysis showed differences for exons 44 (*p* < 0.0001) and 46 (*p* = 0.035, Yate’s corrected), but for the other exons (45, 51 and 53) no differences were found (*p* > 0.05). According to our study, Mexico and Germany are the countries with the highest frequency of potential candidates for the skipping of exons 44 and 46 (see Table 3). Besides, the intra-population analysis also displayed differences; whereas in populations of countries like Belgium, Bulgaria and China, the best target exon for skipping therapy is the exon 51, for Serbia/Montenegro the best target is exon 45 and in other populations like those in Denmark, France and the Netherlands, among others, all five studied exon targets are evenly distributed (Table 4).

**Table 3:** Frequencies of applicable exon skipping targets among populations (inter-population analysis).

Country	Total of Del/Dup	Exon									
		44		45		46		51		53	
		Del/Dup	%	Del/Dup	%	Del/Dup	%	Del/Dup	%	Del/Dup	%
Australia	159	7	4.4	10	6.29	5	3.14	16	10.06	17	10.69
Belgium	39	0	0	2	5.13	0	0	4	10.26	1	2.56
Bulgaria	23	1	4.35	2	8.7	1	4.35	4	17.39	1	4.35
China	491	18	3.67	39	7.94	14	2.85	67	13.65	58	11.81
Denmark	123	8	6.5	12	9.76	7	5.69	14	11.38	9	7.32
France	1829	93	5.08	132	7.22	65	3.55	174	9.51	148	8.09
Germany	95	11	11.58	3	3.16	9	9.47	9	9.47	5	5.26
Greece	178	5	2.81	8	4.49	4	2.25	36	20.22	19	10.67
Hungary	110	2	1.82	6	5.45	2	1.82	13	11.82	7	6.36
Italy	480	34	7.08	27	5.63	28	5.83	40	8.33	37	7.71
Netherlands	581	45	7.75	47	8.09	33	5.68	61	10.5	42	7.23
Portugal	50	1	2	3	6	1	2	4	8	2	4
Romania	62	2	3.23	6	9.68	0	0	12	19.35	8	12.9
Serbia/Montenegro	71	1	1.41	11	15.49	1	1.41	8	11.27	4	5.63
Mexico (this study)	105	18	17.14	12	11.43	10	9.52	11	10.48	11	10.48
*p*-value	-	*p <* 0.0001		*p* > 0.05		*p* = 0.035		*p* > 0.05		*p* > 0.05	

**Table 4:** Comparison of targets for exon skipping by population (intra-population analysis).

Population	Best Target Exon	*p*-Value	Frequency (%)	Second Best Target	Frequency (%)
Australia	N/A	*p* = 0.11	-	N/A	-
Belgium	Exon 51	*p* < 0.01 *	10.26	Exon 45	5.13
Bulgaria	Exon 51	*p* < 0.01	17.39	Exon 45	8.7
China	Exon 51	*p* = 0.02	13.65	Exon 53	7.94
Denmark	N/A	*p* = 0.67	-	N/A	-
France	N/A	*p* = 0.49	-	N/A	-
Germany	N/A	*p* = 0.15	-	N/A	-
Greece	Exon 51	*p* < 0.01	20.22	Exon 53	10.67
Hungary	Exon 51	*p* < 0.01	11.82	Exon 53	6.36
Italy	N/A	*p* = 0.96	-	N/A	-
Netherlands	N/A	*p* = 0.77	-	N/A	-
Portugal	Exon 51	*p* < 0.01 *	8	Exon 45	6
Romania	Exon 51	*p* < 0.01	19.35	Exon 53	12.9
Serbia/	Exon 45	*p* < 0.01	11.27	Exon 51	11.27
Mexico (this study)	N/A	*p* = 0.53	-	N/A	-

* Yate’s corrected *p*-value.

Recently, potential therapeutic options for DMD have emerged; some, such as gene replacement and cell-based therapies would apply for all patients regardless the type of disease-causing mutation. These are, therefore, considered the most promising therapeutic options [8]. Conversely, gene repair based therapies may only be applicable for a subgroup of patients with particular mutations; IRES-induced translation is a novel mechanism by which ribosomes are recruited directly to specific sites within mRNA making possible cap-independent translation due to the presence of an alternative initiation codon [11] (Figure 1D). This strategy would be useful for patients with mutations within the 5' exons of the DMD gene where the main initiation site is affected by a mutation. In addition, it has been estimated that about 13% [17] of all DMD cases may benefit from treatment with Translarna^®^ (Ataluren) an orally-taken compound that targets nonsense mutations. This novel drug, recently received the first conditional global approval by international regulatory agencies as an orphan drug for combatting nonsense mutations in ambulatory DMD patients aged >5 years [20,21] and it has shown a clinical benefit in terms of delayed disease progression in a phase 2b randomized, double-blind, placebo controlled study [17]. Hence, the results presented herein regarding the frequency of nonsense mutations in Mexican-mestizo patients (10.52%) contribute to a better estimation of the potential applicability of this novel compound in our country. 

One of the most widely studied gene repair strategies is ES, in which reading frame restoration can be achieved using antisense oligonucleotides. These agents target specific exons excluding them from the mature mRNA [22]. The theoretical applicability of exon skipping is estimated at 83% of all DMD mutations, with skipping of exon 51 being applicable to 13% of all DMD patients according to an overall comparison based on the Leiden database [19]. It should be noted that these percentages are global estimates of the potential applicability of ES, rather than a comparison of best exon skipping targets among particular populations. Although several studies have shown that *DMD* mutation hotspots are similar worldwide [23,24], the extent and type of the mutations within these hotspots will determine the best targets for exon skipping, and this may differ among populations [25]. A recent study in Vietnamese patients showed that 27% of cases would benefit from exon 51 skipping, followed by the skipping of exon 45 (20%) and exon 53 (18%), whereas other study revealed that only 9.8% of Japanese patients would benefit from exon 51 skipping [24]. Another independent study showed that 24% of Malaysian DMD patients would benefit from the skipping of exon 45 [26]. All these studies suggest that applicability for exon skipping may vary among patients of different ethnic origin in terms of the presence of significant differences among frequencies of patients with “skippable” mutations among countries, these differences are the result of various combinations of deletions that could be repaired by the omission of the same exon. Our results suggest that population differences regarding the applicability of exon skipping exist. Mexico and Germany are the countries with the highest frequency of potential candidates for the skipping of exons 44 and 46, which is different from other populations (*p* < 0.05). Interestingly, when the frequency of cases for all five target exons are compared between Mexico and China, a difference also exists (chi-square = 24.1 degrees of freedom = 4, *p*-value < 0.001) (Table 3). 

It should be noted that deletion/duplication hotspots within the DMD gene are likely to be similar among populations because these regions are a selection of the resulting phenotype (DMD/BMD) [27]; with a major deletion hotspot around exons 45–52 and a minor hotspot around exons 3–19 [27]; conversely, the deletion of exon 16 is not associated to a muscular pathology [28]. On the other hand, it has been suggested by some authors that the distribution and frequency of deletions within the mutation hotspots of the DMD gene can vary as a result of population-specific intronic sequences [23,29] (*Alu* sequences, short tandem repeats, matrix-associated regions, replication origins, microhomology regions) (reviewed in White and den Dunnen 2006) [27] that predispose individuals to preferential deletion breakpoints [23,29]. Nevertheless, most studies have failed in demonstrating such differences and identifying those sequences because of diverse reasons, such as the techniques employed (multiplex PCR, real-time PCR, southern blot) [30], the scarcity of data available from other populations and intrinsic limitations in statistical analyses due to the heterogeneity in the presentation of data among studies. We do not expect bias in the submission of particular variants that may interfere with our results; care was taken regarding the homogeneity of the techniques employed (only MLPA) for the detection of the analyzed variants. Even though the number of submissions of the populations included in our analysis is a limitation of this study. Currently, the origin of deletion/duplication hotspots and breakpoints within the DMD gene is still unclear and the differences in the frequency of candidates for exon skipping reported herein reflect existent differences in the mutation profiles of populations studied. Therefore, as complete gene screening will become more common and gene databases such as LOVD receive more submissions, our knowledge regarding best targets for molecular gene-repair strategies in different populations will be more ample.

In addition to accurate mutation screening in Mexican patients, it is also important to manage patients correctly so that they can be easily recruited for appropriate clinical trials or given new orphan drugs when changes in public health policies worldwide allow. Patient registries and databases have been shown to be effective in facilitating patient recruitment for clinical trials in Japan [24], France and the Netherlands, among others [31]. Our initial experience in Mexico concerning DMD management was recently reported [32,33], so this study should help to provide better healthcare for DMD patients in Mexico, although sustained efforts by researchers, parent’s organizations and the government will still be necessary to achieve successful personalized molecular medicine for DMD patients in Mexico and elsewhere.

## 3. Experimental Section 

### 3.1. Data and Sample Collection 

A cohort of 180 patients was referred to our laboratory between 2010 and 2013; 170 with a clinical diagnosis of DMD and 10 with BMD; of these, 162 DMD and 8 BMD referrals were unrelated patients from the following nonprofit organizations: (i) Asociación de Distrofia Muscular de Occidente A.C. (Jalisco State, West, Mexico); (ii) ENLACE- Distrofia Muscular Duchenne Becker A.C. (Chihuahua State, North, Mexico); and (iii) Sociedad Mexicana para la Distrofia Muscular A.C. (Mexico City, Center, Mexico). Patients’ clinical evaluation included the presence of proximal and/or distal weakness, positive Gowers’ maneuver, age at onset, serum creatine kinase (CK) levels, and family history. Written informed consent was obtained from parents, according to the organization’s ethical guidelines. 

### 3.2. DNA Extraction

Genomic DNA was extracted from peripheral lymphocytes using the CTAB-DTAB method [34]. A Nanodrop ND-1000 spectrophotometer (Thermo Fisher Scientific, Waltham, MA, USA) was used to measure sample concentration and 100 ng of DNA was used to perform MLPA assays.

### 3.3. Mutation Detection Using PM-MLPA

**Figure 3:**
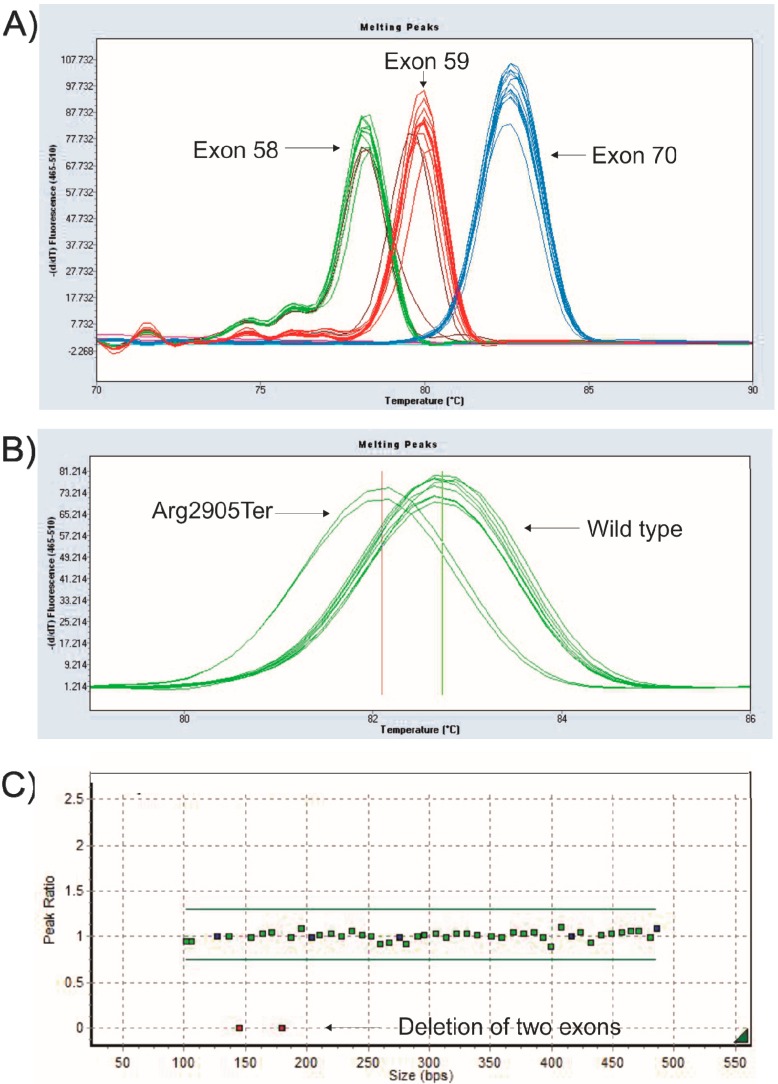
High Resolution Melting (HRM) and MLPA. (**A**) Melting peaks of the three exons that account for most frequent stop codon mutations in the DMD gene; (**B**) Melting peaks of a sample (duplicate) with a point mutation in exon 59 compared to a wild-type sample; (**C**) MLPA assay showing a deletion of two exons of the DMD gene.

Genetic screening for copy number variations of all exons of the DMD gene was done using MLPA according to manufacturer’s instructions (P034/P035, MRC-Holland, Amsterdam, The Netherlands) and analyzed using Genemarker V1.91 software as described previously [35] (Figure 3C). Point mutation specific MLPA probes were applied for the detection of the twenty three most frequent stop codon changes (known variants) in the DMD gene as described previously [2].

### 3.4. Mutation Detection by High Resolution Melting

In order to identify unknown small nucleotide changes, high-resolution melting curves (HRM) were performed on a LightCycler^®^ (Roche, Basel, Switzerland) 480 II platform for 12 exons of the DMD gene (exons 4, 8, 12, 13, 17, 19, 47, 49, 59, 58, 70 and 74) with modifications of the reported protocol [36]. HRM assay for the three most frequently mutated exons are shown in Figure 3A,B. All changes were described using the NG_012232.1 (NM_004006.2) reference sequence and were submitted to the Leiden DMD mutation database. 

### 3.5. DNA Sequencing

Confirmation of point mutations found in the study was done by sequencing, using oligonucleotides reported by Almomani *et al.* 2009 [36]. Purified PCR products were sequenced with Big Dye Terminator v.3.1 (Applied Biosystems, Foster City, CA, USA) according to the manufacturer’s recommendations.

### 3.6. Statistical Analysis

A Chi-square test was performed on the mutation frequencies (correctable deletions/supplications) for the exons that represent targets of current clinical exon-skipping trials (exons 44, 45, 46, 51 and 53). Probability values ≤0.05 were considered significant. All analyses were done using STATGRAPHICS version 16.1.11 (Centurion XVI software, Warrenton, VA, USA). Yates correction was applied if any of the expected values in the calculation were below five.

## 4. Conclusions 

In this study, we described the mutation profile of Mexican-mestizo patients with DMD. Unlike other countries; Germany and Mexico have similar frequencies for the skipping of exons 44 and 46, which were higher than in other populations. As expected, exon skipping of exons 51 and 45 would equally benefit all populations studied, since frequencies do not differ among the countries where the population data is known. Patient registries and gene databases play a pivotal role in the recruitment of patients for novel clinical trials in which multi-ethnic participants from different countries ought to be included in order to improve sample size and statistical power. Mutation detection is of utmost importance for the development of personalized molecular medicine in Duchenne Muscular Dystrophy; our combined PM-MLPA and HRM screening strategy is useful for molecular diagnosis in Mexico, although full-gene sequencing would extend our mutation detection capacity. Finally our data underlie that differences exist in the applicability of exon skipping among particular populations.
